# Dipeptidyl peptidase-4 inhibitory potentials of *Glycyrrhiza uralensis* and its bioactive compounds licochalcone A and licochalcone B: An *in silico* and *in vitro* study

**DOI:** 10.3389/fmolb.2022.1024764

**Published:** 2022-09-30

**Authors:** Sibhghatulla Shaikh, Shahid Ali, Jeong Ho Lim, Hee Jin Chun, Khurshid Ahmad, Syed Sayeed Ahmad, Ye Chan Hwang, Ki Soo Han, Na Ri Kim, Eun Ju Lee, Inho Choi

**Affiliations:** ^1^ Department of Medical Biotechnology, Yeungnam University, Gyeongsan, South Korea; ^2^ Research Institute of Cell Culture, Yeungnam University, Gyeongsan, South Korea; ^3^ Neo Cremar Co., Ltd., Seoul, South Korea

**Keywords:** type 2 diabetes mellitus, dipeptidyl peptidase-4, *Glycyrrhiza uralensis*, natural compounds, licochalcone A

## Abstract

Type 2 diabetes mellitus (T2DM) is a growing global public health issue, and dipeptidyl peptidase-4 (DPP-4) is a potential therapeutic target in T2DM. Several synthetic anti-DPP-4 medications can be used to treat T2DM. However, because of adverse effects, there is an unmet demand for the development of safe and effective medications. Natural medicines are receiving greater interest due to the inherent safety of natural compounds. *Glycyrrhiza uralensis* (licorice) is widely consumed and used as medicine. In this study, we investigated the abilities of a crude water extract (CWE) of *G. uralensis* and two of its constituents (licochalcone A (LicA) and licochalcone B (LicB)) to inhibit the enzymatic activity of DPP-4 *in silico* and *in vitro*. *In silico* studies showed that LicA and LicB bind tightly to the catalytic site of DPP-4 and have 11 amino acid residue interactions in common with the control inhibitor sitagliptin. Protein-protein interactions studies of LicA-DPP4 and LicB-DPP4 complexes with GLP1 and GIP reduced the DPP-4 to GLP1 and GIP interactions, indicated that these constituents might reduce the degradations of GLP1 and GIP. In addition, molecular dynamics simulations revealed that LicA and LicB stably bound to DPP-4 enzyme. Furthermore, DPP-4 enzyme assay showed the CWE of *G. uralensis*, LicA, and LicB concentration-dependently inhibited DPP-4; LicA and LicB had an estimated IC_50_ values of 347.93 and 797.84 μM, respectively. LicA and LicB inhibited DPP-4 at high concentrations, suggesting that these compounds could be used as functional food ingredients to manage T2DM.

## Introduction

The prevalence of diabetes continues to increase rapidly worldwide. Some 463 million people were affected by the disease in 2019, which is expected to increase to 578 million by 2030 and 700 million by 2045 (Saeedi et al., 2019). Moreover, diabetes has been reported to be responsible for around 10% of all fatalities (Reed et al., 2021). Type 2 diabetes mellitus (T2DM) is the most common form of the disease and accounts for ∼90% of cases. T2DM is characterized by inadequate pancreatic insulin production and insulin resistance in peripheral tissues (Chatterjee et al., 2017), and diabetes-related chronic hyperglycemia can result in long-term damage to and the failure of vital organs, such as eyes, kidneys, nerves, and heart (Dodds, 2017; Park et al., 2019).

The term “incretin” refers to a group of hormones that include glucagon-like peptide-1 (GLP-1) and glucose-dependent insulinotropic polypeptide (GIP). When a meal is ingested, these hormones are produced in gut and stimulate insulin production by acting on pancreatic *β* cells (Nauck and Meier, 2018). Dipeptidyl peptidase-4 (DPP-4) degrades circulating GLP-1 and GIP with a half-live of around a minute, and thus, DPP-4 inhibitors enhance active GLP-1 and GIP levels (Pathak and Bridgeman, 2010). Therefore, DPP-4 inhibitors are considered a novel means of treating T2DM. However, currently available DPP-4 inhibitors have several adverse effects such as arthritis, pancreatitis, diarrhea, and congestive heart failure that limit their practical applications (Mascolo et al., 2016; Packer, 2018; Padron et al., 2020), and thus, new DPP-4 inhibitors are needed. Computational techniques had shown to be effective in finding novel drugs and their development in the therapeutic development process (Katsila et al., 2016; Ali et al., 2022).

*Glycyrrhiza uralensis* is a well-known medicinal plant from the Leguminosae family and has been reported to contain various biologically active compounds, which include various triterpene saponins, flavonoids, and licochalcones (Asl and Hosseinzadeh, 2008; Zhang and Ye, 2009; Lee et al., 2021). However, although *G. uralensis* and its biologically active components have been shown to have a variety of pharmacological actions, their effects on DPP-4 inhibition have not been explored. Here, we investigated the ability of the crude water extract (CWE) of *G. uralensis* and two of its bioactive components, namely, licochalcone A (LicA) and licochalcone B (LicB), to inhibit DPP-4 using computational docking simulation analyses and an *in vitro* DPP-4 enzyme assay.

## Methodology

### Protein and ligand preparation

The crystal structure of DPP-4 (PDB ID: 4FFW) was obtained from the Protein Databank and the 3-D structures of LicA (CID: 5318998) and LicB (CID: 5318999) were retrieved from the PubChem database.

### Molecular docking

AutoDock 4.2 was used to dock ligands to DPP-4, and the MMFF94 force field was used to energy minimization of the compounds. Affinity (grid) maps sized 40 × 40 × 40 were generated using an auto grid tool to target grid coordinates in the DPP-4 catalytic site. The x, y, and z values were set at 34.36, 48.98, and 40.19, respectively. The initial positions, orientations, and torsions of ligands were determined randomly. Each docking experiment consisted of 100 separate runs, and each run was programmed to end after a maximum of 2,500,000 energy evaluations. Final figures were produced using Discovery Studio (DS) Visualizer 2021.

### Protein-protein interactions

PatchDock (https://bioinfo3d.cs.tau.ac.il/PatchDock/) was used to run protein–protein docking simulations, which were then refined using FireDock (http://bioinfo3d.cs.tau.ac.il/FireDock/). PatchDock produced 100 predictions for each interaction, which were then submitted to FireDock to select the top ten solutions based on global energy (GE).

### Molecular dynamics simulation

Hidden biological functions and complex mechanisms can be revealed by studying the internal movements of proteins. Molecular dynamics (MD) simulations of DPP4-LicA, DPP4-LicB, and DPP4-sitagliptin were performed using GROMACS (2019.6) at 300 K (Van Der Spoel et al., 2005) using the GROMOS96 43a1 force-field (Pol-Fachin et al., 2009). The PRODRG server was used to generate the compound topology and force-field parameters (Schuttelkopf and van Aalten, 2004). To neutralize ions in the solution, appropriate charges were introduced. To solvate the protein, the Simple Point Charge (spc216) water model was used. The particle-mesh Ewald method was used to investigate interactions between DPP4 and LicA, LicB, and sitagliptin using energy-grps in the MDs parameters (mdp) file. MD systems were then minimized using the steepest descent method (1500 steps) and the NVT and NPT ensembles were used for equilibration. The final production phase (100 ns) was then performed at 300 K. GROMACS analysis modules were employed to examine MD trajectories, and DS 2021 and PyMOL to create graphical representations of 3D models.

### *In vitro* DPP-4 enzyme assay

A DPP-4 Inhibitor Screening Kit (Sigma Aldrich, St. Louis, MO, United States) was used to confirm the inhibitory effects of the CWE of *G. uralensis*, LicA, and LicB *in vitro*. Fluorescence was measured in a microwell plate reader (λexcitation = 360 nm, λemission = 460 nm). Fluorescence emissions were recorded in kinetic mode for 30 min, and relative percentage inhibitions were calculated using the formula below. ΔF/ΔT is the rate of change of fluorescence. All experiments were performed using 6 replicates.

$$\%Relative\;inhibition=\frac{\left(\frac{∆F}{∆T}\right)\;enzyme-\left(\frac{∆F}{∆T}\right)\;enzyme\;inhibitor\;complex\;}{\left(\frac{∆F}{∆T}\right)\;enzyme}\times 100$$

## Results

**FIGURE 1:**
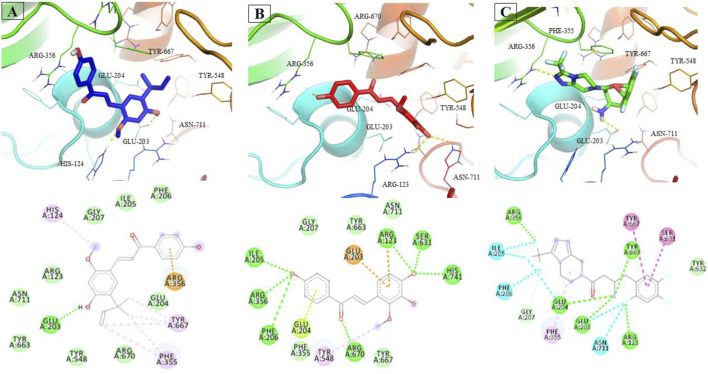
Interacting residues of DPP4 with LicA **(A)** and LicB **(B)**, and sitagliptin **(C)**.

LicA and LicB were found to bind strongly to DPP-4. LicA interacted with the Arg123, His124, Glu203, Glu204, Ile205, Phe206, Gly207, Phe355, Arg356, Tyr548, Tyr663, Tyr667, Arg670, and Asn711 residues of DPP-4, and the Glu203 residue H-bonded with LicA (Figure 1A). LicB interacted with the Arg123, Glu203, Glu204, Ile205, Phe206, Gly207, Phe355, Arg356, Tyr548, Ser631, Tyr663, Tyr667, Arg670, Asn711, and His741 residues of DPP-4, and the Arg123, Ser631, Arg670, and His741 residues H-bonded with LicB (Figure 1B). The binding energies of LicA and LicB with DPP-4 were −6.16 and −6.29 kcal/mol, respectively (Table 1). Further, sitagliptin (the positive control) interacted with the Arg123, Glu203, Glu204, Ile205, Phe206, Gly207, Phe355, Arg356, Ser631, Tyr632, Tyr663, Tyr667, and Asn711 residues of DPP-4 (Figure 1C) with a binding energy of −6.70 kcal/mol (Table 1). ‘Molecular Overlay’ visualization revealed that the binding patterns and conformational alignments of LicA and LicB in the catalytic pocket of DPP-4 were similar to those of sitagliptin (Supplementary Figure S1).

**TABLE 1:** Binding energy of compounds with DPP-4.

Compound	Structure	Binding energy (kcal/mol)	Interacting residues
Licochalcon A	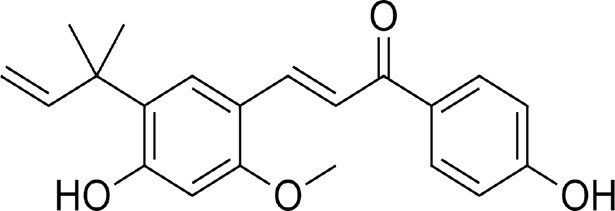	−6.16	Arg123, His124, Glu203, Glu204, Ile205, Phe206, Gly207, Phe355, Arg356, Tyr548, Tyr663, Tyr667, Arg670, and Asn711
Licochalcon B	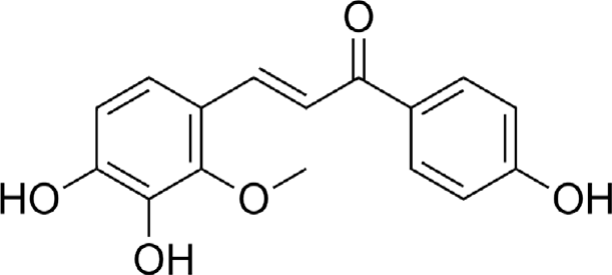	−6.29	Arg123, Glu203, Glu204, Ile205, Phe206, Gly207, Phe355, Arg356, Tyr548, Ser631, Tyr663, Tyr667, Arg670, Asn711, and His741
Sitagliptin	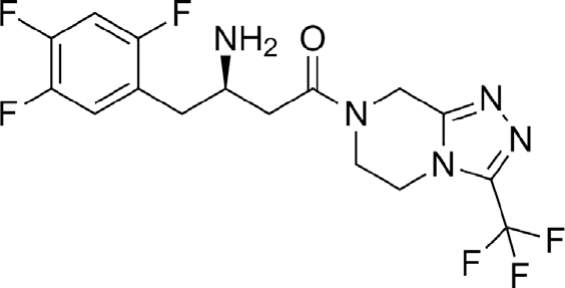	−6.70	Arg123, Glu203, Glu204, Ile205, Phe206, Gly207, Phe355, Arg356, Ser631, Tyr632, Tyr663, Tyr667, and Asn711

**FIGURE 2:**
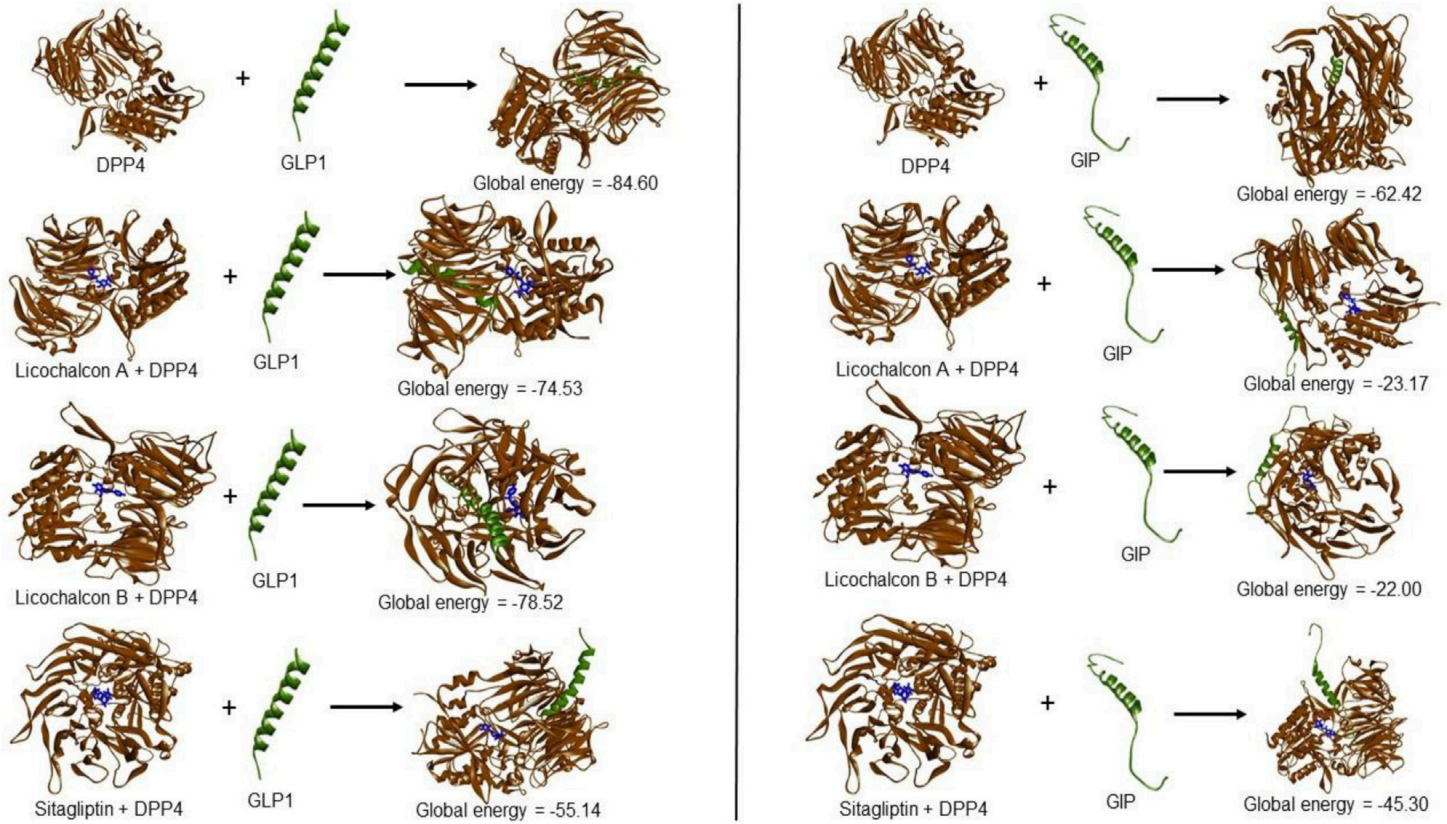
Global energies of LicA, LicB, and sitagliptin DPP-4 complexes against GLP1 and GIP.

LicA-DPP4, LicB-DPP4, and sitagliptin-DPP4 complexes were subjected to PPI with GLP1 and GIP. LicA, LicB, and sitagliptin DPP-4 complexes reduced the DPP-4 to GLP1 and GIP interactions, which suggested LicA, LicB, and sitagliptin might reduce the *in vivo* degradations of GLP1 and GIP (Figure 2). For example, DPP-4 bound to GLP1 with a GE of −84.60, and this was reduced to −74.53, −78.52, and −55.14 in the presence of LicA, LicB, or sitagliptin, respectively. Similarly, DPP-4 bound to GIP with a GE of −62.42, which was reduced to −23.17, −22.00, and −45.30 in the presence of LicA, LicB, or sitagliptin, respectively (Figure 2).

**FIGURE 3:**
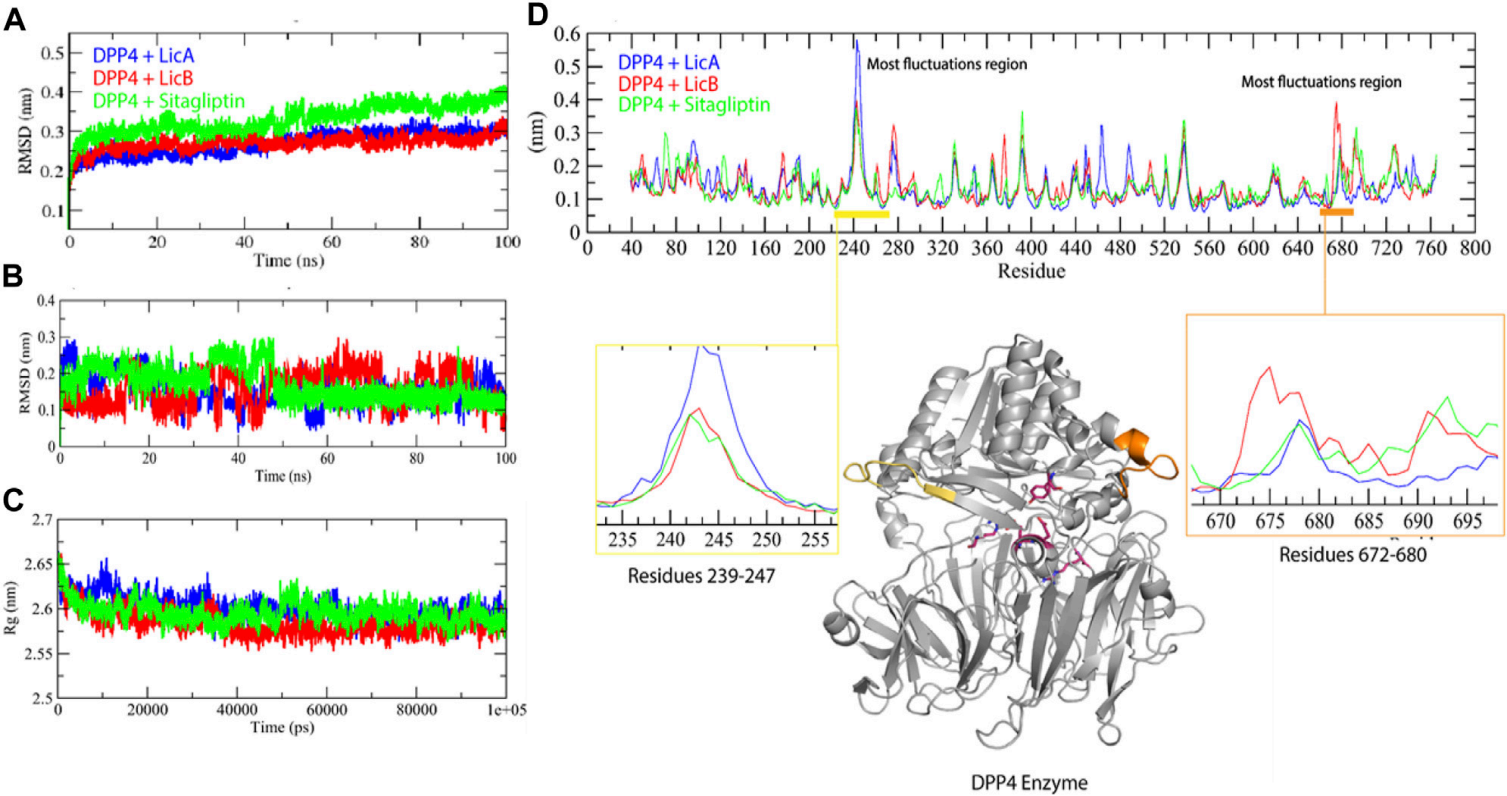
Molecular dynamics simulation studies of DPP-4 with LicA, LicB, and sitagliptin. RMSD backbone of DPP-4 enzyme in complexes **(A)**, RMSD of ligands **(B)**, Rg plot **(C)**, and RMSF plot **(D)**, DDP-4 is shown in gray color.

The stability profiles of LicA, LicB, and sitagliptin DPP-4 complexes were monitored to assess their relative root-mean-square deviation (RMSD) values throughout simulation runs. RMSD is commonly used to infer the extents of spatial deviations of groups of atoms (proteins, ligands, or ligand–protein complexes) from initial reference structure. Here, MD provided RMSD values with respect to the backbone of DPP-4. LicA-DPP4 and LicB-DPP4 complexes had average RMSD values of 0.251 and 0.250 nm, respectively, and sitagliptin-DPP4 complex had a slightly higher average value of 0.335 nm (Figure 3A). We also explored ligand dynamics in the catalytic pocket of DPP4. Visual inspections of complex trajectories revealed that all LicA, LicB, and sitagliptin displayed similar interaction patterns. Interestingly, LicA-DPP4 and sitagliptin-DPP4 complexes had more stable binding in catalytic pocket of DPP-4 (Figure 3B and Movie clip). Further, we assessed the compactness of complexes using the radius of gyration (Rg). The Rg average values for LicA-DPP4, LicB-DPP4 and sitagliptin-DPP4 complexes was found to be 2.59, 2.58, and 2.59 nm respectively. It was determined that there was little difference between the DPP4 enzyme and compound complexes, implying that the enzyme was stable with these compounds (Figure 3C).

Root-mean-square fluctuation (RMSF) plot was used to determine specific residues interacting motion with compounds, and showed that two regions of DPP4, that is, residues 239-247 and 672-680, have a higher fluctuation region. DPP4-LicA complex exhibited fluctuation in the region 239-247, whereas DPP4-LicB complex has high fluctuation in the region 672-680 (Figure 3D). Collectively, these results indicated that DPP4 was stable with binding of LicA, LicB, and sitagliptin.

**FIGURE 4:**
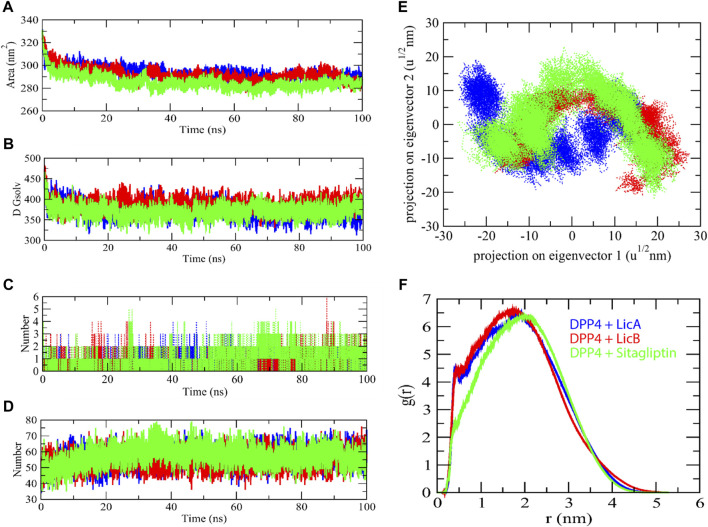
The solvent accessible surface area of complexes. SASA **(A)**, Free energy of solvation **(B)**, Number of H-bond with complexes **(C)**, Number of H-bond between enzyme and water molecules **(D)**, 2D projection of eigenvectors **(E)**, and radial distribution function of complexes **(F)**.

Furthermore, Solvent accessible surface area (SASA) has been assumed to be an important factor in molecular stability and folding studies. The LicA-DPP4, LicB-DPP4, and sitagliptin-DPP4 complexes were reported to have average SASA values of 293.74, 291.87, and 285.53 nm^2^, respectively. The LicA-DPP4, LicB-DPP4, and sitagliptin-DPP4 complexes had solvation energies of 373.21, 387.55, and 370.60 kJ/mol/nm^2^, respectively (Figures 4A,B). The LicA-DPP4 complex showed more SASA than LicB-DPP4 and sitagliptin-DPP4. It was inferred that DPP4 enzyme residues were more expose to water molecules.

H-bond analysis was used to determine the binding interaction pattern of compounds with the DPP4 enzyme. The H-bond is essential for the ligand-protein complex’s stability. The complexes LicA-DPP4 and LicB-DPP4 bind to the DPP4 active pocket with 3–5 H-bonds, whereas sitagliptin-DPP4 binds with 1–3 H-bonds. H-bond analysis with protein and water found that sitagliptin-DPP4 complex had more H-bonds than LicA-DPP4 and LicB-DPP4 complexes (Figures 4C,D). Furthermore, the 2D projections of trajectories on eigenvectors revealed a variety of projections of LicA, LicB, and sitagliptin compounds. There was a significant variation in trajectory projections in the case of the LicA-DPP4 complex. The differences in atom position in LicA are quite distinct from those in LicB and sitagliptin (Figure 4E). This could be due to the fact that different conformations of during MD simulations and radial distribution function revealed that LicA-DPP4 and LicB-DPP4 were more stable than sitagliptin-DPP4 (Figure 4F).

**FIGURE 5:**
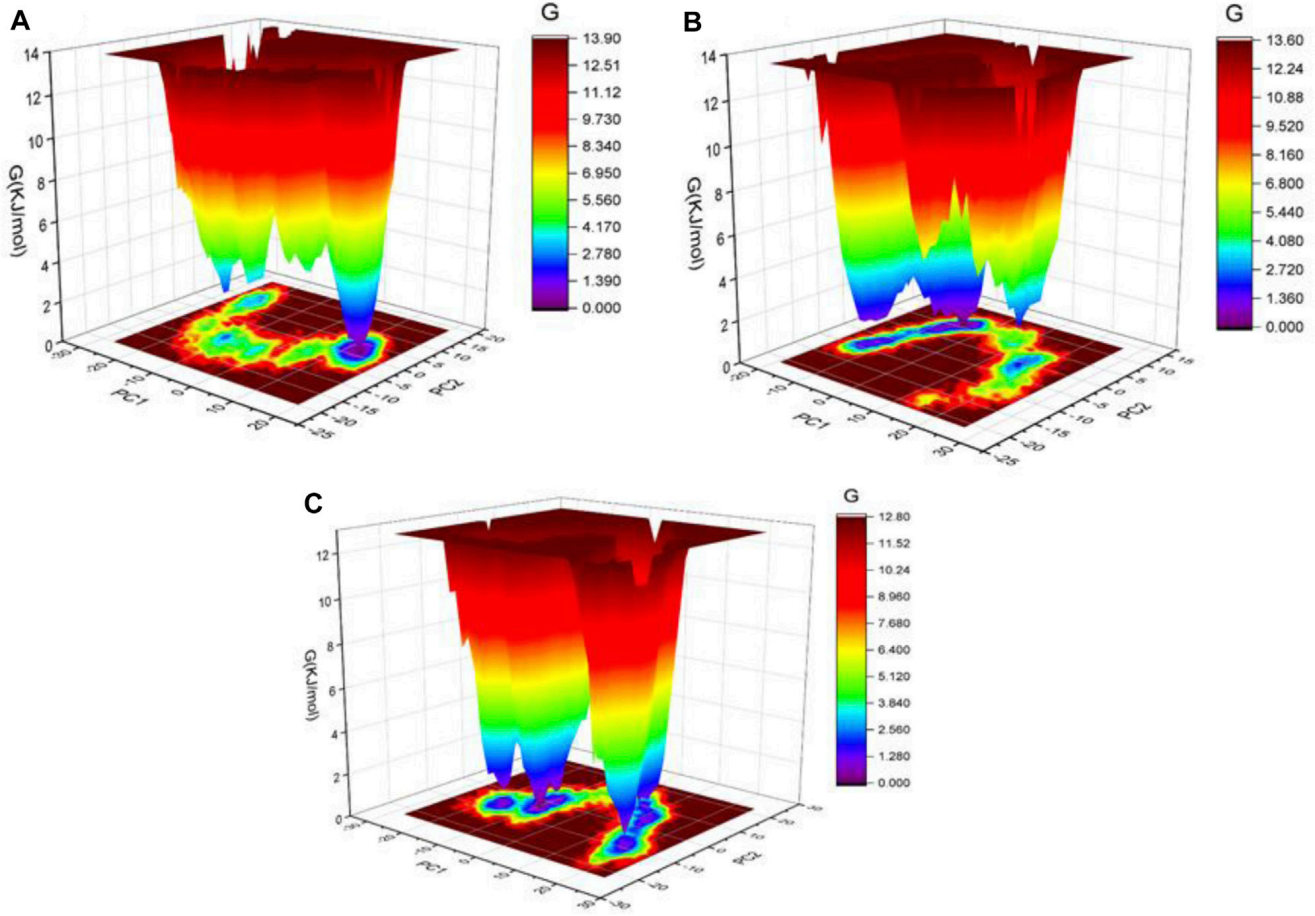
Gibbs’ free energy landscape plot of LicA-DPP4 **(A)**, LicB-DPP4 **(B)**, and sitagliptin-DPP4 **(C)** complexes.

In addition, the Gibbs’ free energy (GFE) landscape was estimated using GROMACS analysis modules and projections of their first (PC1) and second (PC2) eigenvectors. The energy represented by the Comparable GFE contour map with darker blue hues is lower. During the simulations, the complexes interacting with the DPP4 enzyme cause a fluctuation in the global minimum of DPP4. The projections of the LicB-DPP4 and sitagliptin-DPP4 complexes were similar, but the global minima of the LicA-DPP4 complex was different (Figures 5A–C).

**FIGURE 6:**
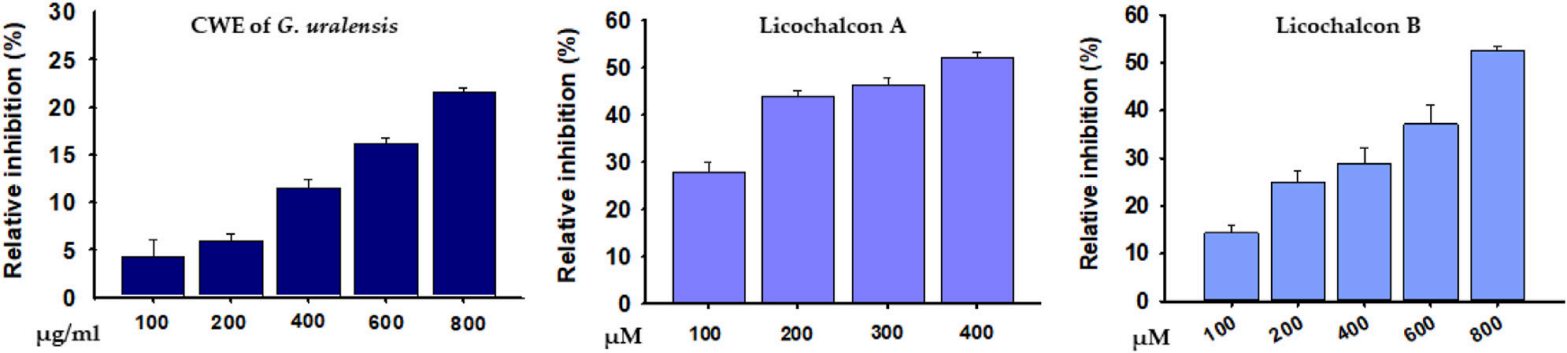
Percentage inhibitions of DPP4 by the CWE of *G. uralensis*, LicA, and LicB.

The abilities of the CWE of *G. uralensis*, LicA, and LicB to inhibit DPP-4 were investigated at different concentrations. At high concentration (800 μg/ml), CWE inhibited DPP-4 by 21.6%. LicA and LicB inhibited DPP-4 activity concentration-dependently with an estimated IC_50_ values of 347.93 and 797.84 μM, respectively (Figure 6).

## Discussion

Licorice root is obtained from perennial herbs native to the Mediterranean, central to southern Russia, and some Asian regions, and the roots of *G. glabra* and *G. uralensis* are commonly used in cosmetics, meals, tobacco, and for several applications in the food and pharmaceutical sectors (Pastorino et al., 2018). Licorice and its bioactive constituents have a variety of beneficial health effects due to their antibacterial, antioxidant, anti-inflammatory, and immunomodulatory activities (Asl and Hosseinzadeh, 2008; Kim et al., 2014; Maria Pia et al., 2019; Wang et al., 2019). This study was performed to assess the possible anti-diabetic effects of the CWE of *G. uralensis*, LicA, and LicB by examining their inhibitory effects on DPP-4.

Molecular docking has become a valuable tool for drug development as it can accurately identify binding modes between drugs and their target proteins (Pinzi and Rastelli, 2019; Ahmad et al., 2021; Lee et al., 2021). In this study, LicA and LicB were found to bind to DPP-4 *in silico*, and an enzyme assay showed that they inhibited DPP-4 *in vitro*. Docking simulation revealed that LicA and LicB inhibit DPP-4 by binding to its catalytic sites. To deep insight into DPP-4 interacting residue with LicA and LicB, we analyzed the DPP-4 residues that interact with its co-crystallized inhibitor sitagliptin (PDB ID: 4FFW) by re-docking sitagliptin with DPP-4 within a defined grid, which revealed that the Arg123, Glu203, Glu204, Ile205, Phe206, Gly207, Phe355, Arg356, Ser631, Tyr632, Tyr663, Tyr667, and Asn711 residues of DPP-4 were involved in sitagliptin binding. Consistent with this, Arg123, Glu203, Glu204, Ile205, Phe206, Gly207, Phe355, Arg356, Tyr663, Tyr667, and Asn711 were the common interacting residues with LicA, LicB, and the sitagliptin. In addition, Glu203 was the common H-bonded residue of DPP-4 with LicA and sitagliptin; while Arg123, and Arg356 were the common H-bonded residues of DPP-4 with LicB and sitagliptin. Overall, our results showed LicA and LicB bind to DPP-4 in the same binding pocket as sitagliptin.

DPP-4 inhibitors are used to treat T2DM because of their ability to increase plasma GLP-1 and GIP levels, and thus, increase insulin production and improve blood glucose control (Deacon, 2020). The degree of interaction between two proteins is assessed in terms of GE, and a high negative GE indicates the strength of the interaction between a protein and its receptor (Lee et al., 2022). We found, DPP-4 interacted with GLP1 with a GE of −84.60, and this was reduced to −74.53 and −78.52 in the presence of LicA and LicB, respectively. On the other hand, DPP-4 interacted with GIP with a GE of −62.42, which was reduced to −23.17 and -22.00 in the presence of LicA, and LicB, respectively. These differences in GEs indicate that LicA and LicB weaken interactions between DPP-4 and GLP1 and GIP and possibly reduce the degradations of these hormones.

The stabilities of LicA, LicB, and sitagliptin DPP-4 complexes were demonstrated by MD simulation, which is a computationally intensive means of simulating the physiological environment of proteins and investigating changes in tertiary structure in biological settings (Adcock and McCammon, 2006). RMSD average values and Rg plots revealed that LicA, LicB, and sitagliptin stably bound to DPP-4 enzyme. Although high fluctuations were observed in some domains of DPP-4 after binding to LicA or LicB, these fluctuations did not occur in DPP-4 binding pocket residues. Furthermore, the MD trajectory movie clip demonstrates that LicA bound to the catalytic pocket of DPP-4 more strongly than LicB, which agrees with our DPP-4 enzyme assay result that LicA inhibits DPP-4 more potentially and has a lower IC_50_ value than LicB.

DPP-4 inhibition is a well-established glucose-lowering treatment in T2DM. Given the various side effects of currently available DPP-4 inhibitors (or ‘gliptins’), which include hypersensitivity reactions, gastrointestinal discomfort, pancreatitis, diarrhea, urinary tract infections, arthritis, and congestive heart failure (Chen et al., 2015; Mascolo et al., 2016; Packer, 2018), the development of natural product-like DPP-4 inhibitors with fewer adverse effects is of considerable importance. Natural products are well-known for providing new molecular entities for treating many diseases (Patridge et al., 2016; Shaikh et al., 2021a; Shaikh et al., 2021b). In addition, phenolics are well known for their ability to alleviate diabetes by lowering blood glucose levels (Rauter et al., 2010). Interestingly, we found that the natural phenols LicA and LicB inhibit DPP-4 activity, and thus, would be expected to have anti-diabetic effects. LicA more potently inhibited DPP-4 with an estimated IC_50_ of 347.93 μM compared to that of the LicB (IC_50_ = 797.84 μM).

The search for natural bioactive compounds in plants has recently been viewed as a priority by the food and pharmaceutical industries due to their commercial potentials (Atanasov et al., 2015). Diet or dietary supplements plays an important role in the prevention and management of diseases such as obesity, diabetes, and cancer (Lee et al., 2013) and increase healthspan (Chen et al., 2022). In this study, LicA, and LicB were found to inhibit DPP-4 at high concentrations, which suggests these agents could be used as functional food ingredients for the management of T2DM.

## Conclusion

LicA and LicB from *G. uralensis* potently inhibit DPP-4 by binding to its catalytic pocket and have several amino acid residues interactions in common with the sitagliptin. MD simulation studies revealed that LicA and LicB bound stably with DPP-4. In addition, DPP-4 *in vitro* enzyme assay showed that LicA, and LicB concentration-dependently inhibited DPP-4. These findings suggest that LicA and LicB may be responsible for the anti-diabetic effects of *G. uralensis* and can be used as functional food ingredients to manage T2DM. Further *in vivo* studies are required to determine whether LicA and LicB can improve glycemic control in animal models of diabetes.

## Data Availability

The original contributions presented in the study are included in the article/Supplementary Material, further inquiries can be directed to the corresponding author.

## Abbreviations

CWE, crude water extract; DPP-4, Dipeptidyl peptidase-4; DS, Discovery studio; GE, Global energy; GFE, Gibbs’ free energy; GLP-1, Glucagon-like peptide-1; GIP, Glucose-dependent insulinotropic polypeptide; LicA, licochalcone A; LicB, licochalcone B; MD, Molecular dynamics; PPIs, Protein-protein interactions; Rg, Radius of gyration; RMSD, Root-mean-square deviation; RMSF, Root-mean-square fluctuation, SASA, Solvent accessible surface area; T2DM, Type 2 diabetes mellitus.
