# Supplementary material for: Dipeptidyl peptidase-4 inhibitory potentials of Glycyrrhiza uralensis and its bioactive compounds licochalcone A and licochalcone B: An in silico and in vitro study
Source: Front Mol Biosci. 2022 Sep 30;9:1024764. doi: 10.3389/fmolb.2022.1024764 (PMC9564220; doi:10.3389/fmolb.2022.1024764)
Supplement: Supplementary file 1 [file Presentation1.PPTX]

## Slide 1
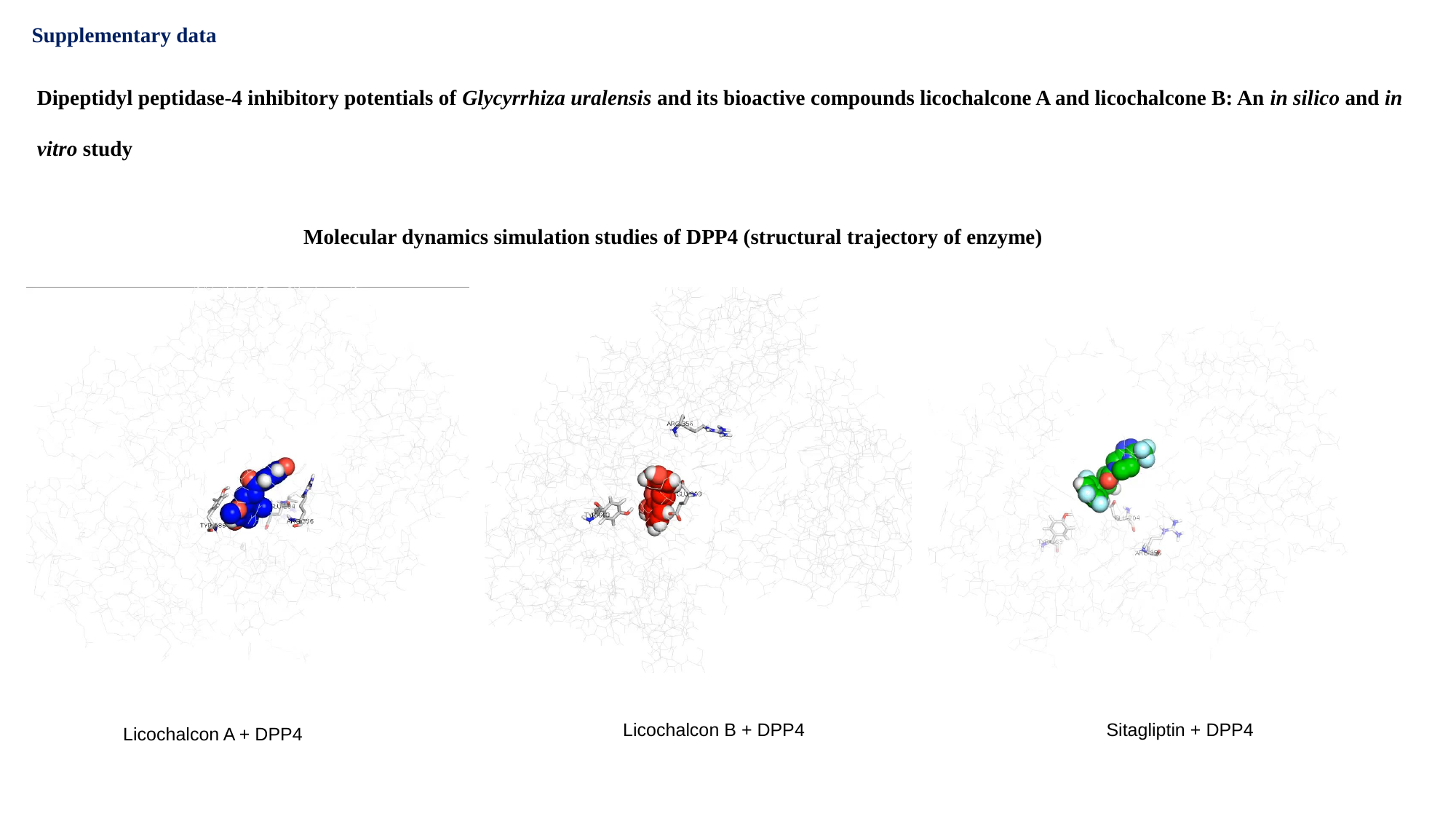

Supplementary data
Dipeptidyl peptidase-4 inhibitory potentials of Glycyrrhiza uralensis and its bioactive compounds licochalcone A and licochalcone B: An in silico and in vitro study
 Molecular dynamics simulation studies of DPP4 (structural trajectory of enzyme)
Licochalcon B + DPP4
Sitagliptin + DPP4
Licochalcon A + DPP4
