# Supplementary material for: Dipeptidyl peptidase-4 inhibitory potentials of Glycyrrhiza uralensis and its bioactive compounds licochalcone A and licochalcone B: An in silico and in vitro study
Source: Front Mol Biosci. 2022 Sep 30;9:1024764. doi: 10.3389/fmolb.2022.1024764 (PMC9564220; doi:10.3389/fmolb.2022.1024764)
Supplement: Supplementary file 2 [file DataSheet1.docx]

**Dipeptidyl peptidase-4 inhibitory potentials of *Glycyrrhiza uralensis* and its bioactive compounds licochalcone A and licochalcone B: An *in silico* and *in vitro* study**

Sibhghatulla Shaikh^1,2†^, Shahid Ali^1,2†^, Jeong Ho Lim^1,2^, Hee Jin Chun^1^, Khurshid Ahmad^1,2^, Syed Sayeed Ahmad^1,2^, Ye Chan Hwang^1^, Ki Soo Han^3^, Na Ri Kim^3^, Eun Ju Lee^1,2*^, Inho Choi^1,2*^

^1^Department of Medical Biotechnology, Yeungnam University, Gyeongsan 38541, Korea

^2^Research Institute of Cell Culture, Yeungnam University, Gyeongsan 38541, Korea

^3^NEO CREMAR Co., LTD, Seoul, 211, Korea


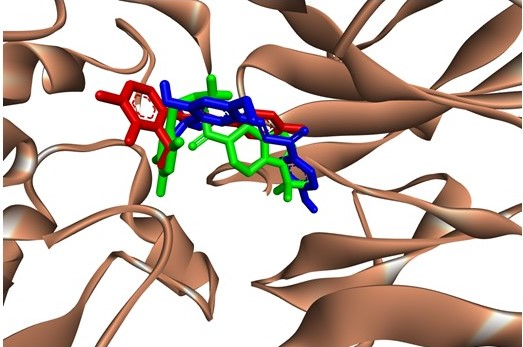


**Figure S1.** ‘Molecular Overlay’ representation of LicA (blue color), LicB (red color), and sitagliptin (green color) in the DPP-4 catalytic pocket.
